# Feasibility of in-bed exercise testing using a clamp-on pedaling device: A single-center pragmatic study

**DOI:** 10.21542/gcsp.2025.63

**Published:** 2025-12-31

**Authors:** Neil K. Jairath, Joshua Mijares, Kishore M. Vora

**Affiliations:** 1 Indiana University Department of Medicine, Indianapolis, IN 46290, USA; 2 Owensboro Medical Practice, Owensboro, KY 42303, USA

## Abstract

Background: Exercise stress testing is the gold standard for evaluating peripheral arterial disease (PAD) and exercise-induced pulmonary hypertension, but traditional treadmill testing has operational limitations including need for patient transport, specialized facilities, and inability to perform invasive hemodynamic monitoring during exercise.

Methods: This prospective single-center feasibility study enrolled 54 consecutive patients undergoing ABI (*n* = 35) or RHC (*n* = 19) procedures at Owensboro Medical Practice. The Bedside Bike (Bedside Bike, LLC; 510(k)-exempt Class I device, product code ION) was integrated into routine vascular laboratory and catheterization laboratory workflows. Primary endpoints included exercise completion rates, data interpretability, and safety. Prespecified success criteria were: completion rate ⩾90% and interpretability ⩾80% for ABI; completion rate ⩾90% and interpretability ⩾70% for RHC; system usability ⩾80% for both.

Results: Exercise was attempted in 94.4% (51/54; 95% CI: 87.0–100%) of patients with a 94.1% (48/51; 95% CI: 86.3–100%) completion rate among those who attempted. Data interpretability was excellent: 97.1% (34/35; 95% CI: 91.4–100%) for ABI and 84.2% (16/19; 95% CI: 68.4–100%) for RHC. All prespecified feasibility criteria were met. System usability ratings were 85.7% (95% CI: 74.3–97.1%) for ABI and 89.5% (95% CI: 73.7–100%) for RHC. Adverse events were minor and occurred in 14.3% of ABI and 5.3% of RHC procedures. Among RHC patients with paired measurements, median cardiac output increased from 4.0 [IQR: 3.8–4.7] L/min at rest to 5.0 [4.8–6.5] L/min with exercise, with a median mPAP/CO slope of 8.26 [6.88–14.12] mmHg/L/min.

Conclusion: This feasibility study demonstrates that bedside exercise testing using a portable pedaling device is technically feasible, safe, and well-tolerated for both ABI and RHC procedures. These preliminary results support larger studies to evaluate diagnostic validity and clinical utility.

## Introduction

Exercise testing has established clinical utility in unmasking cardiovascular pathology that remains subclinical at rest. In peripheral artery disease (PAD), the exercise ankle-brachial index (ABI) serves as the reference standard for diagnosing hemodynamically significant arterial stenoses. When resting ABI values are normal or borderline (0.90–1.40), exercise ABI testing demonstrates superior sensitivity and specificity compared with resting measurements alone^1,2^. Guidelines from the American Heart Association, American College of Cardiology, and Society for Vascular Surgery endorse exercise ABI testing for symptomatic patients with non-diagnostic resting studies^3^. This recommendation reflects the clinical reality that many patients with claudication symptoms demonstrate normal resting hemodynamics but develop characteristic post-exercise pressure declines indicative of flow-limiting disease.

Similarly, in pulmonary vascular medicine, exercise right-heart catheterization (RHC) has emerged as an essential complement to resting invasive hemodynamic assessment, enabling clinicians to assess pulmonary vascular reserve and diagnose exercise-induced pulmonary hypertension. Current guidelines from the European Society of Cardiology and European Respiratory Society acknowledge the diagnostic value of exercise hemodynamics, particularly in patients with unexplained dyspnea, borderline resting pressures, or suspected early pulmonary vascular disease. Exercise RHC unmasks latent pulmonary vascular pathology, facilitates risk stratification, and guides therapeutic decisions across a spectrum of conditions including left heart disease and pulmonary arterial hypertension^4–6^.

### Current limitations and unmet clinical needs

Despite robust scientific rationale and guideline endorsement, exercise testing adoption remains limited across healthcare systems. Multiple operational barriers impede routine implementation, including the need for specialized treadmill facilities, patient transport logistics, fall risk in mobility-impaired populations, and the technical challenge of maintaining invasive hemodynamic monitoring during upright exercise. These constraints disproportionately affect the patients who stand to benefit most: elderly individuals with gait instability, patients with severe claudication limiting treadmill tolerance, and those undergoing invasive procedures in whom simultaneous exercise would provide valuable diagnostic information.

Recent advances in exercise physiology and diagnostic technology have highlighted the potential for supine exercise protocols to provide clinically meaningful diagnostic information while eliminating many traditional barriers. Supine ergometry using a bedside pedaling device can enable exercise testing in bedridden patients, eliminating fall risk, facilitating continuous invasive hemodynamic monitoring, and obviating the need for specialized treadmill facilities. However, adoption of bedside exercise approaches requires demonstration of technical feasibility, workflow integration, hemodynamic data quality, and safety in clinical practice before widespread implementation can be recommended.

### Study rationale and objectives

We conducted a single-center feasibility study to assess whether a bedside pedaling device could be integrated into routine vascular laboratory and catheterization laboratory workflows, informed by established guideline frameworks^1–3,5,6^. Our primary objective was to evaluate feasibility endpoints: exercise attempt and completion rates, data interpretability, system usability, and safety. Secondary objectives included characterizing operational parameters (setup time, additional procedure time) and patient-centered outcomes (comfort, acceptance). This study addresses an important knowledge gap by providing the first systematic evaluation of bedside exercise testing feasibility for both non-invasive (ABI) and invasive (RHC) hemodynamic assessment.

## Methods

### Study design and oversight

We conducted a prospective, single-center, non-blinded, single-arm feasibility study at Owensboro Medical Practice under Institutional Review Board approval (#18124). The primary study objective was to assess the feasibility, safety, and preliminary operational characteristics of bedside exercise testing in patients scheduled for routine ABI assessment or clinically indicated RHC. Prespecified success criteria were: (1) exercise completion rate ⩾90% and data interpretability ⩾80% for ABI procedures; (2) exercise completion rate ⩾90% and data interpretability ⩾70% for RHC procedures; (3) system usability rating ⩾80% (easy or very easy to use) for both procedure types.

The IRB classified this study as minimal risk research, permitting collection of data that clinical staff could record during routine patient care without requiring protected health information beyond standard clinical documentation. Clinical staff entered study data immediately following each case using a structured Google Form interface designed for completion within 60–90 s. Written informed consent was obtained from all participants prior to device use. This manuscript adheres to the Transparent Reporting of Evaluations with Nonrandomized Designs (TREND) statement for reporting of feasibility studies.

### Participant selection and enrollment

We enrolled consecutive adult patients scheduled for routine ABI assessment or clinically indicated RHC. Treating clinicians determined the appropriateness and safety of exercise testing based on standard clinical judgment, patient mobility, hemodynamic stability, and procedural factors. Established clinical contraindications to brief lower-extremity exercise (such as; active chest pain, unstable cardiac arrhythmias, hemodynamic instability) or procedural concerns regarding sterility maintenance and patient positioning (complex catheter or monitoring line configurations) excluded patients from exercise testing. All consent procedures followed IRB-approved protocol requirements.

### Device description, specifications, and regulatory status

**Figure 1.**
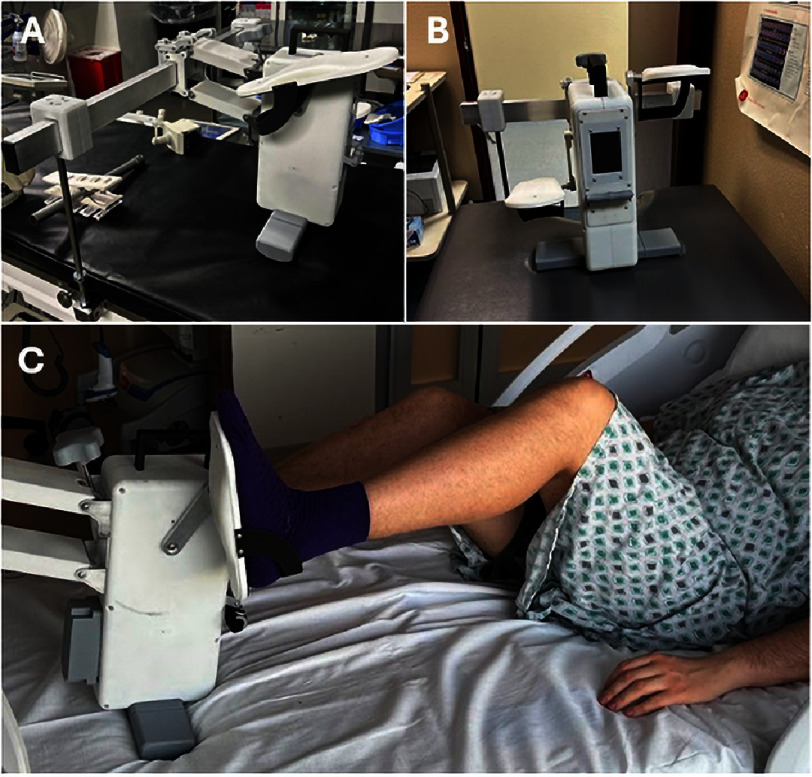
A. Supine exerciser attached to cath lab table. B. Supine exerciser attached to standard outpatient clinic table. C. Patient actively using supine exerciser on a clinic table.

The Bedside Bike device (Bedside Bike, LLC) is a 510(k)-exempt Class I medical device (product code ION, regulation number 890.5370) (Figure 1). The device employs a clamp-on mounting system compatible with standard hospital bed frames, enabling patient-controlled supine pedaling without requiring external power or fixed installation. Adjustable resistance settings allow individualized workload titration. Clinical staff received standardized instruction and supervised hands-on training prior to study initiation. Device cleaning followed institutional infection-control procedures and standard surface disinfection practices for mobile equipment.

### Exercise ABI procedures

Vascular laboratory personnel performed resting ABI measurements according to established local protocols. Following baseline assessments, patients performed supine pedaling for 2–5 min at self-selected intensity while maintained in the standard supine testing position. Clinical personnel monitored patient tolerance and exertion throughout exercise, with early termination permitted at patient request or if clinical concerns arose.

Post-exercise ABI measurements were obtained immediately following exercise cessation, consistent with established protocols emphasizing prompt measurement to detect transient pressure drops characteristic of exercise-induced ischemia^1–3^. Data collection included setup time (⩽60 s, 61–120 s, >120 s), exercise duration, pedaling cadence (slow/moderate/fast), subjective exertion (0–10 scale), measurement timing, post-exercise ABI values, data quality (interpretable both sides/interpretable one side/not interpretable), added procedure time, and system usability ratings by clinical staff.

Safety monitoring documented adverse events (none; cuff/measurement technical issues; patient-initiated early termination; pain or muscle cramping; dizziness or lightheadedness; other complications). Patient-centered outcomes included comfort ratings and willingness to repeat testing.

### Exercise RHC procedures

RHC procedures followed standard institutional protocols for vascular access site selection, sedation administration, and hemodynamic measurement acquisition according to attending physician preference. When clinically appropriate and feasible, patients performed supine pedaling for 2–5 min while hemodynamic monitoring continued, with device positioning to maintain sterile field integrity and avoid interference with catheter position or pressure waveform acquisition.

Staff documentated setup time assessment, exercise duration and intensity, visually estimated pedaling cadence, subjective exertion ratings, staff assessment of exercise waveform interpretability (interpretable/not interpretable), artifact characterization when present, added procedure time relative to standard RHC workflow, sterile field interference assessment, and system usability ratings. When routine clinical care included both resting and end-exercise cardiac output measurements (such as thermodilution techniques), these values were documented in the electronic medical record according to standard practice, enabling exploratory characterization of mPAP/CO slopes^5,6^.

Safety monitoring encompassed comprehensive adverse event tracking (none; transient arrhythmia; hypotensive episodes; patient-initiated early termination; vascular access site disturbance; other complications). Patient comfort and willingness to repeat the procedure if medically indicated were also recorded.

### Outcome measures, statistical planning, and analysis strategy

Primary feasibility endpoints included: setup time distribution (median duration; proportion completing ⩽120 s), additional procedure room time impact (median increase; proportion requiring >5 min), exercise attempt rate (proportion of enrolled patients attempting exercise), exercise completion rate (proportion of attempted procedures completed as planned), data quality (proportion yielding interpretable measurements for clinical decision-making), system usability (proportion of procedures rated easy or very easy by clinical staff), patient comfort (proportion reporting comfortable or very comfortable), patient acceptance (proportion willing to repeat if recommended), and safety (adverse event rate and characterization).

Secondary exploratory endpoints were proposed to include: post-exercise ABI reclassification patterns (proportion of patients with normal/borderline resting ABI demonstrating ≥0.15 decline suggesting hemodynamically significant disease), and exercise hemodynamic response characterization during RHC (paired resting and exercise cardiac output measurements with calculation of mPAP/CO slopes when clinically obtained)^1–3,5,6^. However, systematic collection of pre- and post-exercise ABI values for reclassification analysis was not fully implemented due to workflow constraints, and this planned analysis could not be completed. Exercise hemodynamic data from RHC procedures were available and analyzed as described in the Results section.

We targeted enrollment of 54 consecutive cases (35 ABI procedures, 19 RHC procedures) to provide estimation of feasibility proportions with exact 95% confidence intervals approximating ±10–15 percentage points around point estimates near 90%, adequate for preliminary feasibility assessment. No formal power calculation was performed as this investigation prioritized feasibility assessment rather than hypothesis testing.

Statistical analyses (using Python with scipy package) summarized continuous variables as median [interquartile range] and categorical variables as *n* (%) with exact 95% confidence intervals calculated using the Clopper–Pearson method. Prespecified feasibility success criteria were evaluated by comparing observed point estimates and confidence intervals against predetermined thresholds. Missing data were not imputed; denominators are explicitly reported for each endpoint to ensure transparent interpretation of results. No adjustments for multiplicity were applied given the exploratory nature of this feasibility investigation.

## Results

### Study population and baseline characteristics

A total of 54 patients were enrolled, with 35 (64.8%) undergoing ABI procedures and 19 (35.2%) undergoing RHC procedures. The study population represented a typical cardiovascular patient cohort with median age of 68 (55.6% age ⩾ 65 years), predominantly male (66.7%), with common comorbidities including diabetes mellitus (33.3%), chronic kidney disease (14.8%), and coronary artery disease or heart failure (57.4%). Baseline characteristics are presented in Table 1.

**Table 1:** Baseline demographics and comorbidities (overall cohort).

**Characteristic**	**Overall (*N* = 54)**
Age ⩾ 65 y	55.6%
Male	66.7%
Diabetes mellitus	33.3%
Coronary artery disease or heart failure	50.0%
Chronic kidney disease	18.5%
Known PAD	11.1%
Procedure: ABI	35 (64.8%)
Procedure: RHC	19 (35.2%)

**Notes.**

PAD peripheral artery disease. No PHI collected. CI confidence interval calculated using exact Clopper-Pearson method

### Primary feasibility outcomes

#### Exercise completion rates

Exercise was attempted in 51 of 54 patients (94.4%, 95% CI: 87.0–100%), with 3 patients unable to attempt exercise due to clinical contraindications determined by attending physicians. Of those who attempted exercise, 48 patients successfully completed the procedure as planned (94.1%, 95% CI: 86.3–100%). For ABI procedures specifically, 34 of 35 patients (97.1%, 95% CI: 91.4–100%) attempted exercise with 32 of 34 completing (94.1%, 95% CI: 85.3–100%). For RHC procedures, 17 of 19 patients (89.5%, 95% CI: 73.7–100%) attempted exercise with 16 of 17 completing (94.1%, 95% CI: 82.4–100%). These completion rates met the prespecified success criterion of ⩾90% for both procedure types.

**Table 2:** Operational feasibility and interpretability by procedure.

**Metric**	**ABI (*n* = 35)**	**RHC (*n* = 19)**
Setup time ⩽60 s	77.1%	NR
Setup time ⩽120 s	100%	NR
Added room time (qualitative)	Minimal	Minimal
Exercise attempted	97.1% [91.4–100%]	89.5% [73.7–100%]
Exercise completed (of attempted) [95% CI]	32 (94.1%) [95.3–100%]	16 (94.1%) [92.4–100%]
Data interpretability —overall [95% CI]	97.1% [91.4–100%]	84.2% [68.4–100%]
ABI: both sides interpretable	88.6%	—
ABI: one side interpretable	8.6%	—
ABI: not interpretable	2.9%	—
RHC: not interpretable	—	15.8%
Time from stop to first cuff (ABI)	NR	—

**Notes.**

Interpretable sufficient for clinical reading NR not reported CI confidence interval calculated using exact Clopper-Pearson method

**Table 3:** Patient and staff experience and safety (by procedure).

**Outcome**	**ABI (*n* = 35)**	**RHC (*n* = 19)**
Staff ease-of-use (easy/very easy) [95% CI]	85.7% [74.3-97.1%]	89.5% [73.7–100%]
Patient comfort (comfortable/very comfortable) [95% CI]	88.6% [77.1-97.1%]	73.7% [52.6-89.5%]
Would do again —Yes + Probably [95% CI]	97.1% [91.4–100%]	100% [100–100%]
Adverse events (any) [95% CI]	2 / 35 (5.7%) [2.9-28.6%]	1 / 19 (5.3%) [0-15.8%]
Serious adverse events	0	0

**Notes.**

Comfort and willingness captured via quick verbal check recorded by staff. AE counts reflect the detailed safety paragraph and supersede earlier summaries.

CI confidence interval calculated using exact Clopper- Pearson method

Three patients did not attempt exercise. Patient 1 (ABI procedure; 66-year-old female with coronary disease) presented with recent heart failure exacerbation and NYHA Class III symptoms; attending physician determined exercise would pose unacceptable risk given decompensated status. Patient 2 (RHC procedure; 54-year-old male with coronary disease requiring assistive device for ambulation) demonstrated severe bilateral lower extremity weakness following prolonged ICU admission and was unable to maintain effective pedaling motion; operator elected to complete resting hemodynamic assessment only. Patient 3 (RHC procedure; 64-year-old female with coronary disease, chronic kidney disease, and requiring assistive device for ambulation) had significant hip and knee osteoarthritis limiting range of motion; patient experienced severe discomfort with attempted hip flexion and could not complete pedaling cycle despite assisted positioning. Full operational feasibility and interpretability metrics can be seen in Table 2. And - in Secondary outcomes: These operational metrics demonstrate seamless integration into existing workflow patterns with minimal time burden. Full patient and staff experience metrics can be seen in Table 3.

#### Data quality

Post-exercise data quality was excellent across both procedure types. For ABI procedures, interpretable measurements were obtained in 34 of 35 cases (97.1%, 95% CI: 91.4–100%), meeting the prespecified success criterion of ⩾80%. Of these interpretable studies, 31 procedures (88.6%) yielded bilateral interpretable measurements while 3 procedures (8.6%) produced interpretable data on one side only. For RHC procedures, interpretable exercise waveforms were obtained in 16 of 19 cases (84.2%, 95% CI: 68.4–100%), exceeding the prespecified success criterion of ⩾70% interpretability.

### Secondary outcomes

#### Usability and workflow integration

Clinical staff rated the system as easy or very easy to use in 30 of 35 ABI procedures (85.7%, 95% CI: 74.3–97.1%) and 17 of 19 RHC procedures (89.5%, 95% CI: 73.7–100%), both meeting the prespecified success criterion of ⩾80% favorable usability ratings. Setup time was ⩽60 s in 27 ABI procedures (77.1%) and ⩽120 s in all 35 ABI procedures (100%). These operational metrics demonstrate seamless integration into existing workflow patterns with minimal time burden.

#### Patient acceptance and comfort

Patient comfort levels were high across both procedure types. For ABI procedures, 31 patients (88.6%, 95% CI: 77.1–97.1%) reported being comfortable or very comfortable with the experience. For RHC procedures, 14 patients (73.7%, 95% CI: 52.6–89.5%) reported being comfortable or very comfortable. When asked about willingness to undergo the procedure again if medically recommended, 24 ABI patients (68.6%) responded affirmatively while 10 (28.6%) indicated they probably would, yielding a combined favorable response rate of 97.1% (95% CI: 91.4–100%). For RHC procedures, 13 patients (68.4%) responded definitely yes while 6 (31.6%) indicated probably yes, yielding a combined rate of 100% (95% CI: 100–100%).

### Exercise hemodynamic response (RHC cohort)

Among the 19 RHC procedures performed, paired resting and exercise cardiac output measurements were obtained as part of standard clinical care in all patients completing exercise. Resting cardiac output was 4.1 [IQR: 3.8–4.7] L/min, increasing to 4.7 [4.5–5.9] L/min with exercise, representing a median increase of 14.6% [10.1–28.8%]. The mean pulmonary artery pressure to cardiac output slope (mPAP/CO slope) was calculated as 11.49 ± 8.35 mmHg/L/min, with median 8.26 [6.88–14.12] mmHg/L/min. Eight of 19 patients (42.1%) demonstrated mPAP/CO slopes >3 mmHg/L/min, a threshold associated with pulmonary vascular disease in published literature. These exploratory hemodynamic data suggest that bedside exercise RHC can capture physiologically relevant changes in cardiopulmonary function.

### Safety outcomes

The bedside exercise testing system demonstrated an acceptable safety profile. Adverse events occurred in 5 of 35 ABI procedures (14.3%, 95% CI: 2.9–28.6%) and 1 of 19 RHC procedures (5.3%, 95% CI: 0–15.8%). All adverse events were minor and self-limited. For ABI procedures, events included cuff measurement technical issues (*n* = 3), patient-initiated early termination (*n* = 1), and minor pain or cramping (*n* = 1). For RHC procedures, one patient initiated early termination without hemodynamic compromise or vascular access disturbance. No serious adverse events, falls, arrhythmias requiring intervention, hypotensive episodes, or procedural complications attributable to exercise testing occurred in either cohort.

## Discussion

### Principal findings

This pilot feasibility study demonstrates that bedside exercise testing using a portable pedaling device is technically feasible, safe, and well-accepted by both patients and clinical staff for ABI and RHC procedures. All six prespecified feasibility criteria were met: exercise completion rates of 94.1% for both ABI and RHC procedures exceeded the 90% threshold; data interpretability of 97.1% for ABI and 84.2% for RHC exceeded thresholds of 80% and 70% respectively; and system usability ratings of 85.7% for ABI and 89.5% for RHC both exceeded the 80% benchmark. These results establish the technical and operational feasibility necessary to justify subsequent studies evaluating diagnostic validity and clinical utility.

The excellent data quality achieved (97.1% interpretable ABI data, 84.2% interpretable RHC waveforms) is particularly encouraging given the challenging bedside environment. However, it is important to emphasize that high interpretability rates in this feasibility study do not establish diagnostic equivalence or accuracy compared to traditional testing methods. The seamless workflow integration, with 100% of ABI procedures requiring ⩽120 s setup time and minimal added procedure duration, suggests that adoption barriers related to time constraints and workflow disruption may be manageable in real-world practice settings.

### Feasibility findings and clinical context

The high feasibility demonstrated in this study addresses an important operational barrier to exercise testing in contemporary practice. Traditional exercise ABI protocols require patient ambulation to treadmill facilities, which may be contraindicated in patients with severe claudication, gait instability, or recent revascularization^7^. Bedside exercise testing eliminates these barriers while providing post-exercise pressure measurements that are the diagnostic foundation of exercise ABI interpretation^1–3^. However, this feasibility study did not include direct comparison to treadmill testing or validation of diagnostic accuracy, which would be essential before recommending bedside approaches as clinical alternatives.

For RHC procedures, the 84.2% interpretable waveform rate demonstrates that exercise can be successfully performed during invasive hemodynamic assessment in the majority of patients^9^. Exercise RHC has been shown to provide incremental diagnostic and prognostic value beyond resting measurements alone, particularly in patients with borderline resting pressures or unexplained dyspnea^5,6^. The exploratory hemodynamic data from our cohort, including measurable increases in cardiac output and calculation of mPAP/CO slopes, suggest that bedside exercise can capture physiologically relevant cardiopulmonary responses. Nevertheless, the clinical significance of these measurements requires validation in larger cohorts with standardized protocols and correlation with functional capacity, symptoms, and outcomes.

### Patient-centered outcomes

The high patient satisfaction rates (88.6% ABI, 73.7% RHC comfortable or very comfortable) and near-universal willingness to repeat testing if medically indicated (97.1% ABI, 100% RHC) indicate that bedside exercise testing is acceptable from a patient experience perspective. These patient-centered outcomes are encouraging and suggest that bedside approaches may be preferred by some patients compared to traditional exercise modalities requiring ambulation. The minimal adverse event rate (14.3% ABI, 5.3% RHC) with only minor, self-limited symptoms demonstrates the safety of bedside exercise testing even in patients with significant cardiovascular comorbidities^10^, though longer-term safety monitoring in larger cohorts would be prudent.

### Limitations

This investigation has several important limitations that warrant careful consideration. First and most importantly, this was a feasibility study focused on technical success, safety, and operational characteristics rather than diagnostic validation. We did not include a comparator arm (such as traditional treadmill testing), did not perform systematic validation of diagnostic accuracy, and cannot make claims about clinical equivalence or superiority based on these data. The high data interpretability rates demonstrate technical feasibility but do not establish that bedside measurements provide equivalent diagnostic information to established protocols. Comparative effectiveness studies with direct head-to-head testing and validation against reference standards would be essential before recommending bedside approaches for routine clinical use.

Second, this was a single-center pilot study with a relatively small sample size (54 patients), which limits generalizability to other healthcare settings with different patient populations, staff expertise, and operational workflows. The study population was selected by treating physicians using clinical judgment rather than standardized inclusion/exclusion criteria, introducing selection bias that may overestimate feasibility and safety in unselected populations^8^. Our results may not generalize to centers without similar expertise or to patients deemed unsuitable for exercise by our clinicians.

Third, exercise protocols in this study were not standardized or calibrated in terms of absolute workload, intensity, or duration. Patients exercised at self-selected intensity for 2–5 min, which may not provide equivalent physiologic stress to standardized treadmill protocols. The lack of standardized workload limits comparability across patients and precludes correlation of hemodynamic responses with objective exercise capacity metrics. Absence of quantitative workload measurements also prevents calculation of exercise capacity or comparison to established normative data. As more data is gathered, standardized protocols for supine exercise devices may be created.

Fourth, the planned exploratory analysis of post-exercise ABI reclassification patterns (proportion of patients with normal/borderline resting ABI demonstrating ⩾0.15 decline) could not be completed due to incomplete systematic collection of pre- and post-exercise ABI values across the cohort. This limits our ability to characterize the clinical diagnostic yield of bedside exercise ABI testing. Future studies should prospectively collect paired resting and post-exercise values with standardized timing to enable meaningful assessment of diagnostic reclassification patterns.

Fifth, hemodynamic data obtained during RHC procedures represented opportunistic clinical measurements rather than systematically protocolized research data collection. Cardiac output measurement techniques, timing of measurements, and level of exertion at which measurements were obtained varied according to clinical workflow and physician preference. While these data demonstrate feasibility of obtaining exercise hemodynamic measurements, they should be interpreted as preliminary observations rather than rigorously standardized research findings.

Sixth, the expertise of the clinical team at our institution and the support provided during the feasibility evaluation may have contributed to the positive outcomes observed^8^. Results may differ during initial implementation at other centers without similar training or support. Learning curve effects, institutional factors, and variations in procedural workflows may impact feasibility, data quality, and safety metrics. Multi-center evaluation with diverse institutional characteristics would provide more robust feasibility assessment.

Finally, this study did not evaluate cost-effectiveness, comparative healthcare resource utilization, long-term patient outcomes, or downstream clinical decision-making impacts. Whether bedside exercise testing leads to clinically meaningful diagnostic information, appropriate therapeutic decisions, or improved patient outcomes remains unknown and represents a critical knowledge gap that must be addressed in subsequent investigations.

### Future directions

These encouraging feasibility results support several important next steps for advancing bedside exercise testing. A larger, multi-center validation study comparing bedside exercise testing to traditional treadmill protocols would provide essential diagnostic accuracy data and establish the clinical utility necessary for guideline consideration. Such studies should include standardized exercise protocols with quantitative workload measurement, systematic collection of paired resting and post-exercise measurements, blinded interpretation of results, and correlation with clinical outcomes.

Future research should also explore optimization of exercise protocols for different patient populations, development of standardized training programs for clinical staff, evaluation of learning curve effects across diverse institutional settings, and health economic analysis of implementation costs and resource utilization impacts. Randomized controlled trials evaluating whether bedside exercise testing influences clinical decision-making, therapeutic interventions, and patient outcomes would ultimately determine the clinical value proposition beyond technical feasibility.

## Conclusions

This single-center feasibility study demonstrates that bedside exercise testing using a portable pedaling device is technically feasible, safe, and well-tolerated for both ABI and RHC procedures. All prespecified feasibility criteria were met, with high completion rates, excellent data interpretability, favorable system usability ratings, and an acceptable safety profile. These preliminary findings establish the foundational feasibility necessary to justify larger validation studies. However, it is essential to emphasize that demonstration of technical feasibility does not establish diagnostic validity, clinical equivalence to traditional methods, or clinical utility. Subsequent comparative effectiveness studies with rigorous diagnostic validation are required before bedside exercise approaches can be recommended for routine clinical implementation.

## Acknowledgements

The dedicated support staff of nurses and technicians at Owensboro Medical Practice for their unwavering commitment to patient care.

## Author Contributions

NJ, JM, and KV contributed to study design, execution, data analysis, data collection, and writing and manuscript review. KV contributed facilities and oversight.

## Statements and Declarations

Not applicable.

## Ethical Considerations

IRB Approved, Owensboro Medical Practice #18124. Written informed consent obtained from all participants.

## Conflict of Interest

The author(s) declared no potential conflicts of interest with respect to the research, authorship, and/or publication of this article. Bedside Bike, LLC had no involvement in study design, data collection, analysis, or manuscript preparation.

## Funding statement

No external funding received for this study.

## Patient Consent

Written informed consent was obtained from each patient.
